# Extravasation of Urine, Followed by Extensive Sloughing of the Scrotum, Integuments of the Penis and of the Abdomen; Successfully Treated by Laying Open the Perineum, and by Free Incisions

**Published:** 1827-09

**Authors:** 

**Affiliations:** St. Thomas's Hospital.


### EXTRAVASATION OF URINE.

Case I. *Extravasation of Urine, followed by extensive Sloughing of the Scrotum, Integuments of the Penis and of the Abdomen; successfully treated by laying open the Perineum, and by free Incisions.* Treated by Mr. Green, at St. Thomas's Hospital.

September 21st, 1826.—Stephen Casey, æt. thirty-seven, sailor and corn-porter, was admitted, with tenderness, swelling, and induration of the bulb of the urethra. He stated that he had led an irregular life, had drank freely, but had generally enjoyed good health; that for seventeen years he had had a stricture, which had followed an attack of gonorrhœa. About three months ago, he again contracted gonorrhœa, for which he was admitted into the hospital-ship off Greenwich; when sarsaparilla was administered for about three weeks, and he was then dismissed nearly well. A gleety discharge continuing to annoy him, he became a patient in Guy's Hospital, where he remained only a short time. Three or four days prior to his admission into St. Thomas's, he was much exposed in a damp situation, and was attacked with severe rheumatic pains in the limbs, and with tenderness and swelling in the perineum.

Applicentur Hirud. x. ad perinæum, et postea Catap. Lini.

22d.—There is considerable febrile excitement of the system. The pulse is quick and hard; tongue foul; bowels confined. Has passed water freely. There is slight tenderness in the course of the spermatic cord.

℞. Calomel, Pulv. Opii, āā gr. j. M. hàc nocte sumend.—℞. Magnes. Sulphat. ʒj.; Vin. Antim. Tartarizat. m. xv. è Mist. Magnes. Carb. sextâ quâque horâ sumend.—Fever diet.

23d.—Increased swelling of the perineum, accompanied by dark red unhealthy inflammation of the scrotum, in the course of the spermatic vessels, and across the lower part of the abdomen. Bowels still confined.

℞. Pulv. Scammon. c. Calomel gr. xv. statim sumend.—Applic[r] Lotio Spir. Vini.—Milk diet.

25th.—Has passed no urine since yesterday. The scrotum is much inflamed, mottled, and vesicated; as are also the integuments of the penis. The inflammation extends in the course of the cord to the lower part of the abdomen, which is of a vivid red colour, and also vesicated, but not in a state of gangrene. From these appearances, it was evident that the urethra had given way, and that the urine was extravasated; and, in order to obviate further mischief from the injection of the urine into the cellular membrane, an incision was made in the course of the raphè of the perineum into the urethra, when a quantity of very fetid urine, mixed with pus, escaped; the scrotum was then freely divided, to allow the more ready separation of the sloughs. It was also proposed to incise the integuments of the penis, but to this measure the patient so strenuously objected, that it was not done.

℞. Pulv. Ipecac. comp. gr. v. ex Mist. Potass. Citrat. sextâ quâque horâ sumend.—Catap. Lini.—Fever diet.

26th.—Has passed an anxious, restless night. Pulse frequent, small, and sharp; tongue white and dry; skin hot; considerable thirst; bowels not confined. The greater portion of the scrotum and the integuments on the dorsum penis are in a state of slough. The whole of the hypogastric region is vividly inflamed and vesicated: to this part the spirit lotion is still applied.

The patient presented similar constitutional symptoms for several days. On the 27th, Pulv. Opii gr. j. was administered at night; and the following day a similar quantity was directed to be taken thrice daily, on account of his extreme restlessness and irritability. The inflammation did not extend beyond the limits already pointed out, and gradually became circumscribed as suppuration and sloughing of the cellular membrane occurred in each groin.

29th.—An incision was made through the thinned integument in the right groin, and exit allowed for a quantity of pus and dead cellular membrane. The pulse is frequent and soft; the bowels are freely open; the tongue is white and moist, and a gentle perspiration exudes from the surface. There is less restlessness and irritability, but he complains of want of sleep.

October 2d.—Cerev. ℔j. quotidie. The integuments in the left groin have given way under the ulcerative process, and a copious discharge of pus escaped; the surface of the ulcer is covered by dead cellular membrane; as is also that in the right groin, where the ulceration is still extending. The sloughs are loosening here, and also from the scrotum and dorsum of the penis.

8th.—The patient has become much emaciated, from the very profuse discharge. The bowels are relieved twice or thrice daily; the appetite begins to return.

Mutton-chop alternate days. Porter Oij. daily; port wine ℥ ij. with Sago and Syrup, daily. To stimulate the ulcers, and quicken the separation of the sloughs, apply Catap. Cerevis.

12th.—The sloughs have nearly separated from the scrotum and penis; the ulcers in the groin are clean, but irritable, the integument being undermined; that in the right groin is as large as the palm of a moderate-sized hand; that in the left as large as a half-crown piece. The bowels are relaxed; appetite good; pulse 110, small and feeble; tongue rather white. Is very restless, and has very little sleep.

14th.—Repeat Pulv. Opii gr. j. sextâ quâque horâ.

20th.—The sloughs have all separated, and the ulcers are healthy; the discharge is still copious. General health improving.

Granulation and cicatrisation rapidly proceeded; and, as the discharge diminished in quantity, the patient gradually recovered his strength and bulk. On the 25th October, it was ascertained that a stricture existed at the lower part of the scrotum, and, to remedy this complaint, a metallic catheter was directed to be passed occasionally, but not to be worn. The granulations becoming luxuriant, the red precipitate ointment was applied on November 10th.

November 27th.—On account of irregularity and improper behaviour in the ward, he was discharged; the wounds being nearly healed, excepting that in the perineum, whence the urine still flowed.

